# PCR pattern of HIV-exposed infants in a tertiary hospital

**DOI:** 10.11604/pamj.2014.18.345.3713

**Published:** 2014-08-28

**Authors:** Onankpa Ben, Tahir Yusuf

**Affiliations:** 1 Department of Paediatrics, Usmanu Danfodiyo University Teaching Hospital, Sokoto, Sokoto State, Nigeria

**Keywords:** PCR pattern, HIV-exposed, tertiary hospital, Nigeria

## Abstract

**Introduction:**

Early infant diagnosis (EID) provides a critical opportunity to strengthen follow-up of HIV-exposed children and early access to antiretroviral treatment. The study is designed to determine PCR pattern of HIV- exposed infants.

**Methods:**

A 2-year cross-sectional study at Usmanu Danfodio University Teaching Hospital (UDUTH), Sokoto, Nigeria. All pregnant women that presented to our ANC between January, 2011 and December, 2012 were screened for HIV; confirmation for seropositivity was from a positive ELISA and then a Western Blot assay. PCR was done for all the HIV-exposed babies at 6-8 weeks of age. Statistical analysis was done using SPSS version 20.0.

**Results:**

Otal delivery was 6,578. One hundred and sixty three babies from 162 mothers were HIV-exposed; 88 males, 75 females, with male to female ratio of 1.2:1. Eighty eight (54.0%) of the mothers were on HAART before pregnancy; 63 (39.0%) commenced HAART during pregnancy while, 12 (7.0%) never received HAART. Three (1.8%) of the HIV-exposed babies had a positive PCR. One hundred and thirty nine babies (85.3%) were breast fed.

**Conclusion:**

Mother-to-child-transmission of HIV appears to be on the decline in the study area (1.8%), this probably, represents the pattern in other parts of the country.

## Introduction

Nigeria has the third largest number of people living with HIV worldwide, with about 3 million Nigerians infected [1–3]. Prevalence of HIV infection among pregnant women visiting antenatal clinic is about 4.4% with mother-to-child transmission of 10% [1–3]. According to the recommendations of the World Health Organization (WHO), infants known to have been exposed to the human immunodeficiency virus (HIV) should undergo a virological test for infection at 4 to 6 weeks of age [4, 5]. Antiretroviral therapy (ART) should be initiated upon diagnosis of HIV infection in children aged less than 24 months [6]. However, implementing programmes for such early infant diagnosis and treatment has proved challenging [7]. HIV-infected children are most vulnerable of all patients. In infants who acquire HIV at the time of delivery, disease progresses rapidly in the first few months of life, often leading to death [8, 9]. The exposed child who receives prophylactic antibiotics (Cotrimoxazole) and anti-retroviral therapy (ART) as soon as is medically indicated, has a significant chance of long and healthy survival [8, 9]. Despite the increase in effective methods to prevent mother-to-child transmission of human immunodeficiency virus type 1 (HIV-1), there were an estimated 390,000 new pediatric HIV-1 infections in 2010, the majority of which occurred in resource-limited settings [10, 11]. The overall treatment coverage of children also remains poor as only 23% of the HIV-infected children estimated to need antiretrovirals currently have access to treatment [11]. Throughout Africa, the diagnostic challenge of HIV-exposure in infants is being addressed by scaling up virological testing using dried blood spots (DBS) for polymerase chain reaction (PCR) [11]. Available data suggest that only 8% of infants born to women with HIV infection receive a virological test within the first 2 months of life. [10–12]. We therefore carried out this study to determine HIV reactivity using EID and then link the reactive infants to appropriate care, support, and treatment.

## Methods

This was a cross-sectional study (prospective) carried out at paediatric ART clinic of Usmanu Danfodiyo University Teaching Hospital (UDUTH), Sokoto, Nigeria. The study population comprised all perinatally HIV exposed children aged six weeks to eight weeks; Dried Blood Spot (DBS) samples were taken for a DNA PCR test between January, 2011 and December, 2012. All the pregnant women who registered for antenatal care in the study facility received HIV testing and counseling. Following the National PMTCT guidelines at the time of the study, pregnant women who tested positive for HIV were evaluated for clinical and immunological eligibility for the commencement of ART [13]. Eligible mothers were commenced on a first line highly active antiretroviral therapy (HAART) regimen which consisted of Zidovudine (AZT), Lamivudine (3TC) plus either Nevirapine (NVP) or Efavirenz (EFZ). The choice of the specific regimen was guided by the clients′ clinical condition and presence of contra-indication to any of the drug options. All clients had adherence counseling before initiating HAART and on every other visit for drug refill or replacement. Nevirapine (NVP) was given to babies born to HIV positive women within the first 72 hours of life and then continued for six weeks. The HIV-exposed infants were thereafter screened for HIV according to the National EID algorithm which stipulates that a DNA PCR test should be carried out on all HIV-exposed infants at age six weeks. HIV-exposed infants were initiated on cotrimoxazole prophylaxis and their caregivers advised to return in two weeks to receive the DNA PCR test results. The test was carried out using the GeneAmp PCR System 9700 (Perkin-Elmer, Norwalk, Conn, USA). Infants with a positive HIV result were counseled thereafter, enrolled into our facility HIV care and treatment services, while those who breastfed and those with a negative HIV results were advised to return for a repeat DNA PCR test six weeks after the complete cessation of breastfeeding. Ethical approval was obtained from our hospital ethical committee. Data collected was sorted out manually for accuracy and consistency, and entered into SPSS version 20.0 for statistical analysis. Frequency counts were performed to assess completeness of all variables. The DNA PCR result was the major outcome variable in this study and was determined for mother-baby pairs. Statistical significance was set at p value < 0.05. Differences in proportions were tested using Chi-square test.

## Results

The total delivery for the study period was 6,578. Table 1 showed the gender distribution and the PCR result of the exposed infants; one hundred and sixty three babies from one hundred and sixty two mothers (a mother had a set of twins) were HIV-exposed; 88 were males, 75 were females with male to female ratio of 1.2:1. Three (1.8%) of the HIV-exposed babies had a positive PCR i.e. one (1) baby of a mother that was commenced on HAART during pregnancy, two (2) babies of mothers who never had HAART during pregnancy while, no baby was positive for mothers who were on HAART before pregnancy, (x^2^= 0.53, p < 0.47). Eighty eight (54.0%) of these mothers were on HAART before pregnancy, 63 (39.0%) commenced HAART during pregnancy while, 12 (7.0%) mothers never received HAART during pregnancy, p < 0.0001 (Table 2). The distribution of PCR result of the exposed infants based on feeding options is shown in Table 3; one hundred and thirty nine babies (85.3%) including the 3 positive ones for HIV (positive PCR) were breastfed (24 babies had mixed feeding, 139 babies were exclusively breastfed, p < 0.36). Fifty one babies out of the 163 HIV-exposed babies had a second PCR done for them i.e. including the 3 babies that had a positive PCR.

**Table 1:** Gender distribution of PCR result of HIV-exposed infants (N = 163)

Results	Male	Female	Total
Positive	1	2	3
Negative	87	73	160
Total	88	75	163

X^2^ = 0.53 p < 0.47

**Table 2:** Time of commencement of maternal HAART and outcome of PCR in HIV-exposed babies (N = 163)

Maternal HAART	Positive	Negative	Total (%)
None (16.7%)	2	10	12
Before Pregnancy (0.0%)	0	63	63
During Pregnancy (1.6%)	1	87	88
Total	3	160	163

X^2^ = 16.02 p < 0.0001

**Table 3:** Distribution of PCR results based on feeding options of HIV-exposed infants (N = 163)

Feeding option	Positive	Negative	Total
Mixed feeding	2	23	25 (8.7%)
Breastfeeding	1	136	138 (0.7%)
Total	3	159	163

X^2^ = 0.84 p < 0.36

## Discussion

The prevention of mother-to-child transmission (PMTCT) of HIV programme was officially commenced in study area in 2008 with the collection of DBS from HIV-exposed babies which were sent to kano, Nigeria (Regional Head Area for IHVN) for PCR analysis. The Institute of Human Virology, Nigeria (IHVN) however installed a PCR machine in the study area in 2010, since then all our PCR analysis is done in the study centre. Our PMTCT programme involves effective prevention of vertical transmission of HIV between a mother and her baby i.e. an acceptance of voluntary HIV counselling and testing, receiving ARV prophylaxis in positive mothers and safe infant feeding practices. Data from our earlier PCR analysis of 16 months in 2008/2009 showed a 21.2% positive PCR in babies of 52 mothers who were positive for HIV (two-third of the mothers then were not on ARVs and babies received only sdNVP) [14]. The present study however, revealed a lower value of 1.8% in 163 mothers in 24 months (i.e. after four years of the commencing the PMTCT programme). This change is more likely an indication that our PMTCT programs were better implemented over time; the proportions of mothers accessing efficacious regimens had increased and, mothers and infants receiving no ARVs or sdNVP only had decreased significantly. The HIV transmission rate of 1.8% observed for babies at six weeks in this study though, relatively higher, compared reasonably with related studies on HIV transmission rates in PMTCT program settings. In Zambia [2, 15], it was observed that the transmission rates among babies between zero and six weeks old was 6.5%. Similarly a study in South Africa by Mnyani et al recorded an overall transmission rate of 5.8% for HIV exposed babies between four and six weeks of age, where both mother and baby received some form of ARVs for PMTCT [16].

The finding that one hundred and thirty nine (85.3%) of the exposed babies screened for HIV in this study were breastfed, is consistent with a similar study in Zambia where about 84% of HIV exposed infants were breastfed [15]. But, HIV transmission rates of babies who were formula fed showed minor changes over time compared to an observed higher HIV transmission rates for babies who had mixed feeding; this suggests that exclusive breastfeeding and/or formula feeding alone is safer than mixed feeding as a feeding option for HIV exposed infants. This is consistent with previous facts from the ZVITAMBO project in Zimbabwe, which demonstrated that compared with early breastfeeding, early mixed feeding was associated with a four-fold risk of HIV transmission at six months of age for HIV-exposed babies who had previously tested negative at six weeks of age [2, 17]. Our further analysis also indicated that the HIV positive rates were higher in babies that their mothers never received ARVs during pregnancy (16.7%) or commenced ARVs only during pregnancy (1.6%) compared to the mothers that started ARVs before pregnancy; and, this was also consistent with previous studies from Zambia with a value of 20.1% of transmission rate in mothers without ARVs during pregnancy [18]. Our study has shown how analysis of routine program data can be a useful tool for assessing the outcome of a PMTCT program and has also highlighted how MTCT is significantly reduced when ARVs are used before and during pregnancy. The use of ARVs still remains the cornerstone of PMTCT interventions and combined ARVs offer a better reduction of MTCT.

**Limitations:** our study has limitations for the fact that this is a hospital based study and therefore some of the HIV exposed children in the community will not show up for EID. These babies might be either infected and/or died thus, the MTCT rate that our study reports might only be an underestimation of the true MTCT rate in the study area. A larger community-based study is required and also we were not able to do a repeat PCR for all the babies.

## Conclusion

Centers with Paediatric ART clinic should improved client tracking and strengthening the link between the PMTCT, EID and the paediatric ART. Proper counseling of the mothers of HIV-exposed babies will increase the uptake and retaining of these babies in ART programs.
